# Glycemic variability and in-hospital death of critically ill patients and the role of ventricular arrhythmias

**DOI:** 10.1186/s12933-023-01861-0

**Published:** 2023-06-12

**Authors:** Yuhao Su, Weiguo Fan, Yang Liu, Kui Hong

**Affiliations:** 1 Department of Cardiovascular Medicine, The Second Affiliated Hospital of Nanchang University, No.1, Minde Road, 330006 Nanchang, Jiangxi China; 2 Jiangxi Key Laboratory of Molecular Medicine, Nanchang, Jiangxi China; 3 Department of Genetic Medicine, The Second Affiliated Hospital of Nanchang University, Nanchang, Jiangxi China

**Keywords:** Blood glucose, Glycemic variability, Ventricular arrhythmias, In-hospital death

## Abstract

**Background:**

Abnormal glycemic variability is common in the intensive care unit (ICU) and is associated with increased in-hospital mortality and major adverse cardiovascular events, but little is known about whether adverse outcomes are partly mediated by ventricular arrhythmias (VA). We aimed to explore the association between glycemic variability and VA in the ICU and whether VA related to glycemic variability mediate the increased risk of in-hospital death.

**Methods:**

We extracted all measurements of blood glucose during the ICU stay from The Medical Information Mart for Intensive Care IV (MIMIC-IV) database version 2.0. Glycemic variability was expressed by the coefficient of variation (CV), which was calculated by the ratio of standard deviation (SD) and average blood glucose values. The outcomes included the incidence of VA and in-hospital death. The KHB (Karlson, KB & Holm, A) is a method to analyze the mediation effect for nonlinear models, which was used to decompose the total effect of glycemic variability on in-hospital death into a direct and VA-mediated indirect effect.

**Results:**

Finally, 17,756 ICU patients with a median age of 64 years were enrolled; 47.2% of them were male, 64.0% were white, and 17.8% were admitted to the cardiac ICU. The total incidence of VA and in-hospital death were 10.6% and 12.8%, respectively. In the adjusted logistic model, each unit increase in log-transformed CV was associated with a 21% increased risk of VA (OR 1.21, 95% CI: 1.11–1.31) and a 30% increased risk (OR 1.30, 95% CI: 1.20–1.41) of in-hospital death. A total of 3.85% of the effect of glycemic variability on in-hospital death was related to the increased risk of VA.

**Conclusion:**

High glycemic variability was an independent risk factor for in-hospital death in ICU patients, and the effect was caused in part by an increased risk of VA.

**Supplementary Information:**

The online version contains supplementary material available at 10.1186/s12933-023-01861-0.

## Background

Stress hyperglycemia is common in the intensive care unit (ICU) due to the effects of blood glucose-regulating hormones (glucagon, catecholamine, growth hormone and cortisol) [1]. Hyperglycemia was associated with increased in-hospital mortality and long-term mortality. However, recent studies have indicated that intensive insulin therapy increases mortality, which is possibly due to hypoglycemic events and significant fluctuations in blood glucose [2]. Thus, in addition to hyperglycemia, high glycemic variability could also be a risk factor for adverse prognosis.

Glycemic variability is defined as the fluctuation of blood glucose measurements within a given interval of time, which is the main pattern of abnormal blood glucose in critically ill patients [3]. Many factors induce significant glycemic variability in ICU patients, including older age, stress hyperglycemia, enteral or parenteral nutrition, and medications (steroids, adrenaline and insulin) [4, 5]. Higher glycemic variability is associated with new-onset atrial fibrillation in patients with diabetes and mortality within 1 year in heart failure patients without diabetes [6, 7]. Damage of acute abnormal blood glucose metabolism to the cardiovascular system has been confirmed by a series of studies, which could lead to severe ventricular arrhythmia (VA) including ventricular tachycardia (VT) and ventricular fibrillation (VF) [8–10]. It was reported that hyperglycemia, hypoglycemia and high glycemic variability could prolong the corrected QT interval and dispersion in electrocardiogram, which is linked to polymorphic VT [9, 11]. Sympathetic activation induced by abnormal blood glucose is also a risk factor for fatal VA and mortality [8]. Sustained VA were detected in 2% of patients admitted to general ICUs, and their in-hospital mortality was as high as 73% [12]. Although observational studies have indicated that abnormal glycemic variability is associated with in-hospital mortality or major adverse cardiovascular events, little is known about whether adverse outcomes are partly mediated by VA [5, 13]. We performed a retrospective study to explore the correlation among glycemic variability, VA and in-hospital death to apply some available information to improve the management of blood glucose and VA in the ICU.

## Methods

### Database source

The Medical Information Mart for Intensive Care IV (MIMIC-IV) database is a publicly available clinical database that includes patients who were admitted to the ICU of Beth Israel Deaconess Medical Center between 2008 and 2019 [14]. An approved researcher Yang Liu (certification number: 55,302,712) extracted the study data, and the code for data query and extraction is available from the MIMIC Code Repository (https://github.com/MIT-LCP/mimic-code).

### Data extraction and study population

We defined the first day of ICU admission as baseline. The demographics (sex, age and race), ICU category, vital signs, simplified acute physiology score II (SAPS II), laboratories and comorbidities were extracted. The comorbidities were identified by ICD-9 or ICD-10 codes of discharge diagnoses, and the details are shown in Supplemental Table 1.

### Exposure

For patients with repeat ICU admission, we only enrolled the data of the first. We extracted all measurements of blood glucose during the ICU stay to evaluate glycemic variability, and patients with fewer than three measurements were excluded. Additionally, patients with a length of ICU stay less than 24 h or more than 30 days were excluded. We calculated the coefficient of variation (CV) to represent glycemic variability. The CV was the ratio of SD and average blood glucose of all repeat measurements.

### Ventricular arrhythmia and in-hospital mortality

**Fig. 1:**
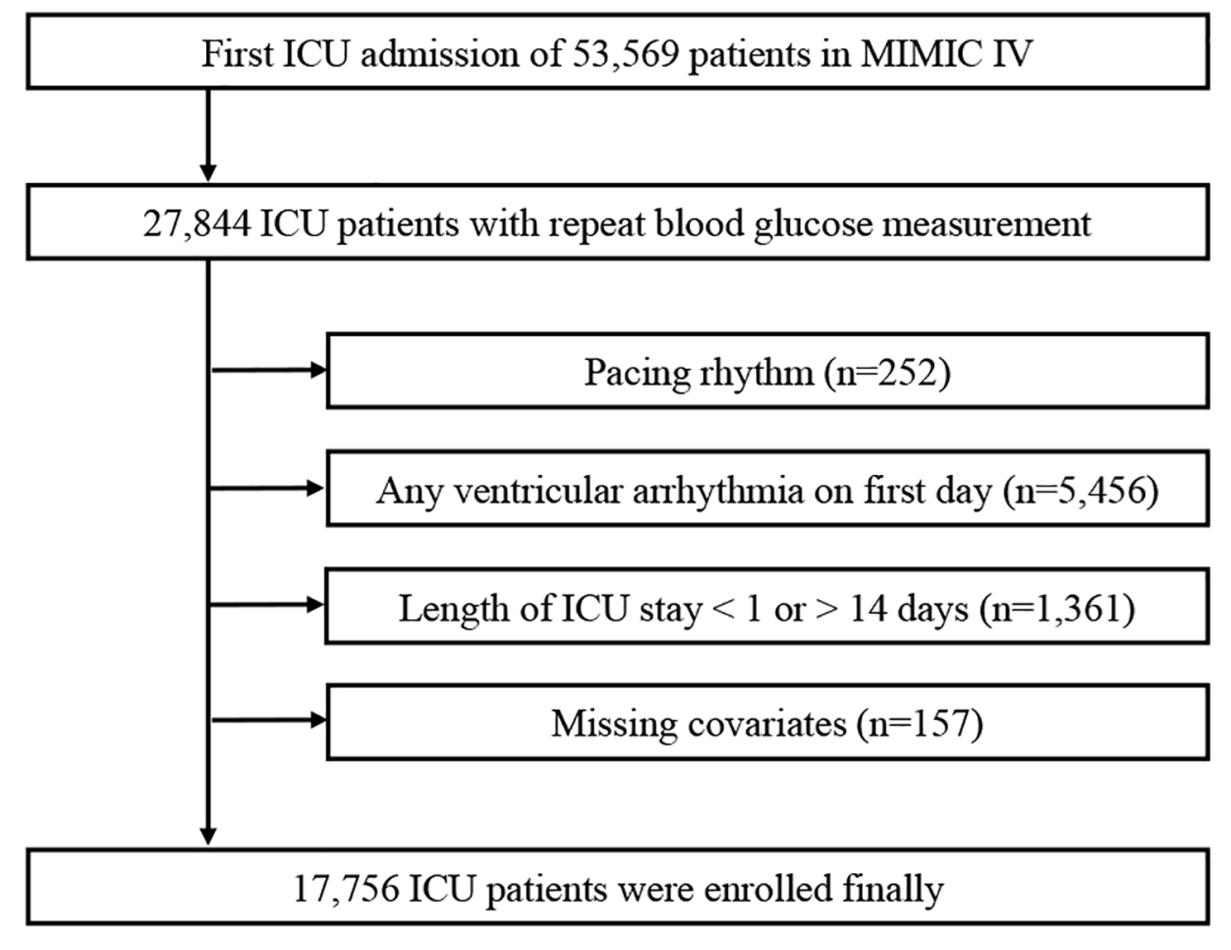
Flow chart of the inclusion of study population

In the MIMIC-IV database, the heart rhythm information was stored in an event table. Primary rhythm, frequency of ectopy rhythm and time were recorded in detail. Patients missing heart rhythm information, pacing rhythm or any VA at baseline were excluded (Fig. 1).

The outcomes included the incidence of VA and in-hospital death. Occasional premature ventricular contractions (PVC) were common during continuous ECG monitoring, and the clinical significance was slight; thus, the PVC described as occasional were not the outcomes of this study. The VA in this study included frequent PVC, non-sustained VT, sustained VT and VF. In-hospital death was determined by the date of death and discharge.

### Statistical analyses

Continuous variables are expressed as the mean ± SD or median and interquartile range (IQR), and the baseline characteristics among groups were examined by one-way analysis of variance or Kruskal‒Wallis test, respectively. The frequency distribution of continuous variables was shown in histogram in Supplemental Fig. 1, and the results of normality test were shown in Supplemental Table 2. Categorical variables are expressed as numbers and percentages (%), and differences were examined by Fisher’s exact test.

Because of the nonnormal distribution of CV, natural log-transformed CV was used for analysis as a continuous variable and three groups by tertiles of CV. We used crude and adjusted logistic regression models to determine the association between glycemic variability and outcomes. The covariates included age, sex, race, heart rate, mean arterial pressure, SAPS II, laboratory results (eGFR, potassium, and sodium), comorbidities (heart failure, myocardium infarction, cerebrovascular disease, diabetes, chronic pulmonary disease, sepsis and tumors), and mean blood glucose during ICU admission.

We assessed whether the association between glycemic variability and in-hospital mortality was mediated by VA. KHB could estimate coefficients between two nest nonlinear probability models, which allows separation of the change in the coefficient that is due to confounding and the change that is due to rescaling, additionally allows other confounding variables to be adjusted [15]. The KHB method was used to decompose the total effect of glycemic variability on in-hospital death into direct and indirect effects (mediated by VA). Finally, we performed subgroup analyses based on the preset groups, including age (< or ≥ 65 years), sex, diabetes, and ICU categories. Moreover, a four knots (P25, P50, P75, P95) restrict cubic spline (RCS) was used to show possible non-linear association between glycemic variability and in-hospital mortality.

All statistical analyses were performed by Stata version 17 (Stata Corp., College Station, TX), and a P value less than 0.05 was considered statistically significant.

## Results

A total of 17,756 ICU patients with a median age of 64 years were enrolled; 47.2% of them were male, 64.0% were white, and 17.8% were admitted to the cardiac ICU. 2,266 (12.8%) patients died in hospital. 1,877 patients (10.6%) experienced one or more episodes of VA. Notably, the incidence of frequent PVC, non-sustained VT and sustained VT/VF were 8.43%, 3.28% and 0.74%, respectively. The baseline characteristics according to tertiles of CV were shown in Table 1.

**Table 1:** Baseline characteristics of study population according to tertiles of glycemic variability

Characteristics	Total patients n = 17,756	T1 (< 14.0%) n = 5,919	T2 (14.0-23.4%) n = 5,919	T3 (> 23.4%) n = 5,918	P value
Age,	64 (52–76)	63 (50–76)	65 (51–76)	65 (53–76)	< 0.001
Male, n (%)	8,372 (47.2)	2,001 (45.1)	2,050 (46.2)	2,152 (48.5)	0.001
Race, White	11,364 (64.0)	2,933 (66.1)	2,882 (64.9)	2,852 (64.3)	< 0.001
Black	1,748 (9.8)	490 (8.3)	536 (9.1)	722 (12.2)	
Hispanic/Latino	666 (3.8)	224 (3.8)	188 (3.2)	254 (4.3)	
Asian	603 (3.4)	178 (3.0)	208 (3.5)	217 (3.7)	
Other/unknown	3,375 (19.0)	1,155 (19.5)	1,124 (19.0)	1,096 (18.5)	
ICU, Cardiac	3,165 (17.8)	758 (17.1)	755 (17.0)	777 (17.5)	0.002
Other	14,591 (82.2)	3,681 (82.9)	3,684 (82.3)	3,662 (82.5)	
Heart rate	86 (75–98)	84 (73–96)	86 (75–98)	87 (75–99)	< 0.001
Systolic BP	117 (107–130)	120 (108–133)	118 (107–131)	116 (106–129)	< 0.001
Diastolic BP	63 (57–71)	65 (58–73)	64 (57–72)	63 (56–70)	< 0.001
Mean arterial pressure	78 (71–87)	80 (73–89)	78 (72–87)	77 (71–85)	< 0.001
Comorbidities					
Myocardial infarction	2,487 (14.0)	487 (11.0)	575 (13.0)	625 (14.1)	< 0.001
Congestive heart failure	3,975 (22.4)	783 (17.6)	910 (20.5)	1,066 (24.0)	< 0.001
Chronic pulmonary disease	4,124 (23.2)	924 (20.8)	982 (22.1)	1,098 (24.7)	< 0.001
Cerebrovascular disease	3,040 (17.1)	904 (20.4)	847 (19.1)	688 (15.5)	< 0.001
Diabetes	4,986 (28.1)	691 (15.6)	890 (20.1)	1,237 (27.9)	< 0.001
Sepsis	9,972 (56.2)	1,919 (43.2)	2,499 (56.3)	2,699 (60.8)	< 0.001
Malignant cancer	2,742 (15.4)	643 (14.5)	706 (15.9)	717 (16.2)	0.127
Charlson comorbidity index	5 (3–7)	5 (3–7)	5 (3–7)	5 (3–8)	< 0.001
Laboratories					
Potassium, mEq/L	3.8 (3.5–4.2)	3.8 (3.5–4.2)	3.8 (3.5–4.2)	3.8 (3.5–4.2)	0.056
Sodium, mEq/L	137 (134–140)	138 (135–140)	137 (134–140)	137 (134–140)	< 0.001
eGFR, ml/min/1.73m^2^	59 (33–89)	68 (45–96)	63 (38–92)	57 (31–88)	< 0.001
Initial blood glucose, mg/dL	129 (106–166)	117 (102–135)	126 (107–150)	137 (107–171)	< 0.001
Mean blood glucose, mg/dL	126 (109–154)	115 (102–131)	121 (108–139)	128 (112–154)	< 0.001
SAPS II	34 (26–44)	31 (23–39)	33 (26–42)	35 (27–45)	< 0.001
Length of ICU stay	3.2 (2.2–5.2)	2.8 (2.0–4.0)	3.5 (2.3–5.8)	3.6 (2.3-6.0)	< 0.001

### Glycemic variability was associated with VA and in-hospital death

**Fig. 2:**
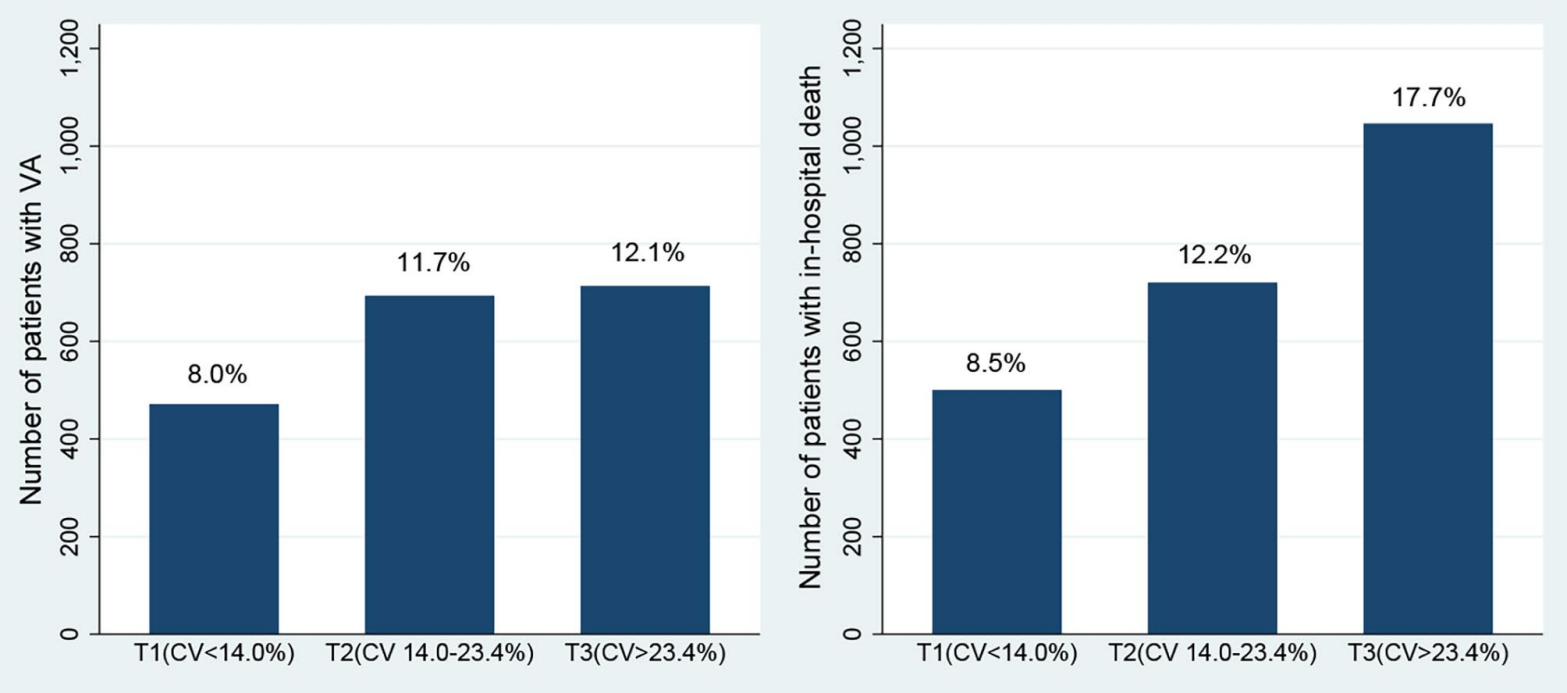
The incidence of VA and in-hospital death among three groups according to tertiles of CV.

In the present study, ICU patients were divided into three groups from tertiles of CV(< 14.0%, 14.0-23.4%, > 23.4%, respectively), based on glycemic variability. The incidences of VA and in-hospital death were 8.0% and 8.5% in patients with lower glycemic variability (CV < 14.0%), 11.7% and 12.2% in those with medium glycemic variability (CV 14.0-23.4%), 12.1% and 17.7% in those with higher glycemic variability (CV > 23.4%), respectively (Fig. 2). Log-transformed CV was positively associated with the risk of VA (OR 1.35, 95% CI: 1.26–1.45, P < 0.001) and in-hospital death (OR 1.74, 95% CI: 1.63–1.87, P < 0.001).

**Fig. 3:**
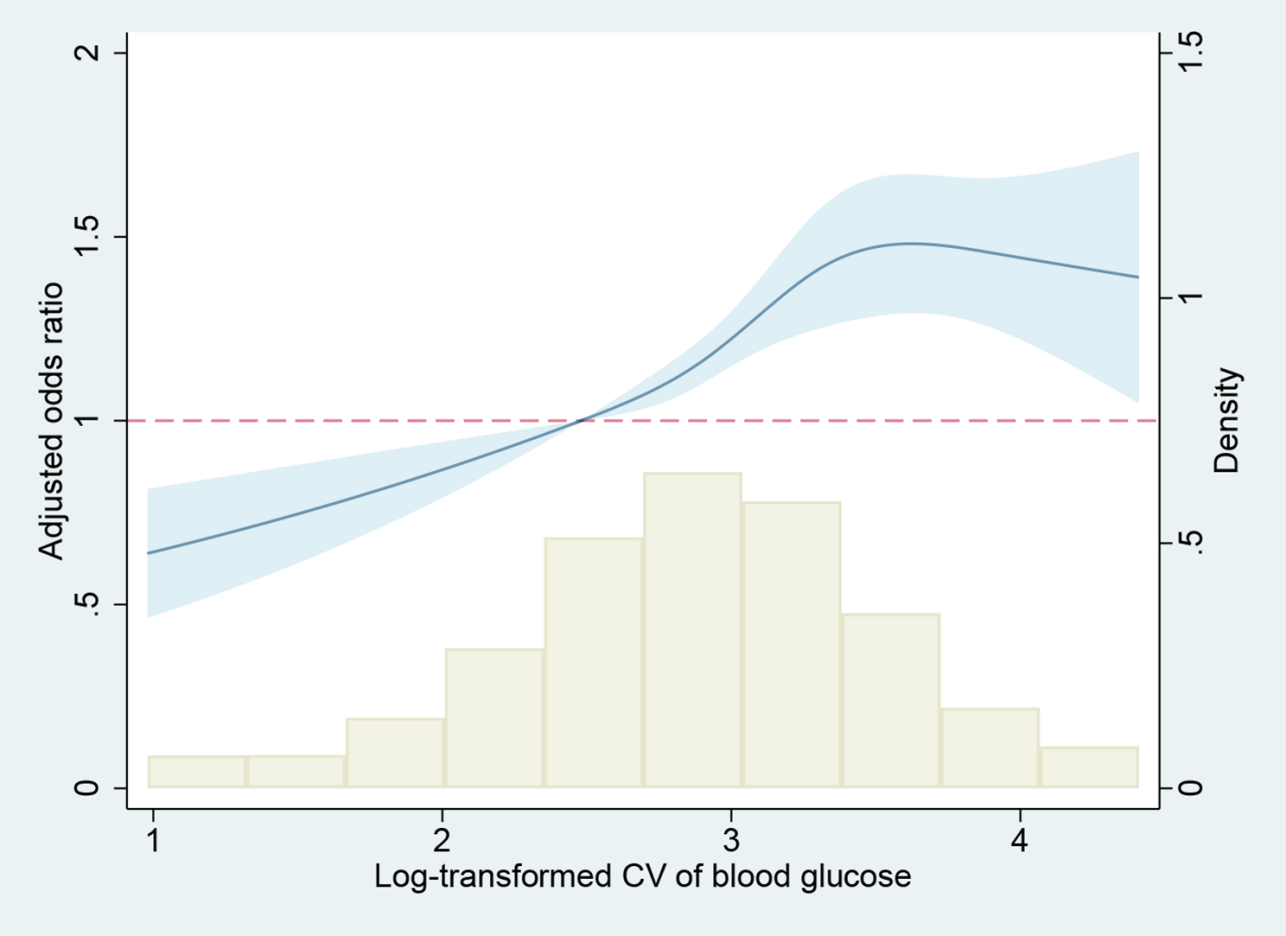
Restricted cubic spline plots for the association of glycemic variability with in-hospital death

An adjusted logistic model was performed to reduce the effect of confounding factors. Controlled variables included age, sex, race, heart rate, mean blood glucose, mean arterial pressure, SAPS II, laboratory results (eGFR, potassium, and sodium), and comorbidities (heart failure, myocardium infarction, cerebrovascular disease, diabetes, chronic pulmonary disease, sepsis and tumors). The detailed results of multivariable logistic regression for VA and in-hospital death were shown in Supplemental Fig. 2 and Supplemental Fig. 3. In the full adjusted logistic model, per unit increase in log-transformed CV was finally found to be associated with a 21% increased risk of VA (OR 1.21, 95% CI: 1.11–1.31) and a 30% increased risk (OR 1.30, 95% CI: 1.20–1.41) of in-hospital death (Table 2). The RCS showed a linear correlation between glycemic variability and in-hospital death (Fig. 3).

**Table 2:** Logistic regression for the association of glycemic variability with VA and in-hospital death

Outcomes	Crude OR Per unit of Log CV	P value	Adjusted OR Per unit of Log CV	P value
VA	1.35 (1.26–1.45)	< 0.001	1.21 (1.11–1.31)	< 0.001
In-hospital death	1.74 (1.63–1.87)	< 0.001	1.30 (1.20–1.41)	< 0.001
VA and in-hospital death	1.75 (1.52–2.02)	< 0.001	1.32 (1.12–1.56)	0.001

Adjusted for age, sex, ICU category, race, sepsis, myocardial infarction, heart failure, cerebrovascular disease, chronic pulmonary disease, malignant cancer, eGFR, serum sodium, potassium, mean arterial pressure, hear rate and average blood glucose

**Table 3:** Direct and indirect effect (mediated by VA) of glycemic variability on in hospital death

	Full association, OR (95% CI)	Direct association, OR (95% CI)	Mediated by VA, OR (95% CI)	Percent mediation, %	P value for indirect effect
Crude	1.76 (1.64–1.89)	1.72 (1.60–1.84)	1.03 (1.02–1.03)	4.41	< 0.001
Adjusted	1.30 (120 − 1.41)	1.29 (1.12–1.40)	1.01 (1.01–1.02)	3.85	< 0.001

Adjusted for age, sex, ICU category, race, sepsis, myocardial infarction, heart failure, cerebrovascular disease, chronic pulmonary disease, malignant cancer, eGFR, serum sodium, potassium, mean arterial pressure, hear rate and average blood glucose

### VA mediated the association between glycemic variability and in-hospital death

We added VA as an independent variable into the mediation model based on logit regression. The effect of glycemic variability on in-hospital death was related to the increased risk of VA, and the proportion of indirect effect by VA was 4.41% in the crude model (P < 0.001) and 3.85% in the adjusted model (P < 0.001).

### Subgroup analyses

Subgroup analyses were performed to verify the stability of the study results. For the association between glycemic variability and study outcomes, no significant interactions were found in subgroups of race, diabetes diagnosis, cardiac or noncardiac ICU, and initial blood glucose level. However, the association between glycemic variability and in-hospital death was more significant in patients aged < 65 years than in those older (P for interaction = 0.012).

## Discussion

In this retrospective study based on medical data from the ICU, we found that increased glycemic variability was associated with VA and in-hospital death, and the effect of glycemic variability on in-hospital death was achieved partly through an increased risk of VA. The results of this study indicated that VA were a possible explanation for the mechanism by which abnormal blood glucose leads to poor prognosis in the ICU.

Before our work, only one study with a small sample enrolled 215 medical ICU patients, but there was no identified correlation between hyperglycemia and VA [16]. Our study enrolled 17,756 ICU patients without any VA on the first day admitted to the ICU, and the results showed that high glycemic variability increased the risk of VA, even after adjusting for known confounding factors and mean blood glucose. Previous studies performed only in the general diabetic population indicated the relationship between glycemic variability and cardiac arrhythmia. In elderly diabetic patients, abnormal glycemic variability increased the susceptibility to atrial fibrillation and VA [17]. Large cohort studies enrolled patients with type 2 diabetes, and glycemic variability was linked to VA and sudden cardiac death [10, 18, 19]. Anderson and colleagues [19] conducted a meaningful study using an implantable cardiac monitor to detect arrhythmic events in patients with type 2 diabetes and found that cardiac arrhythmia were associated with short-term glycemic variability but were not associated with hypoglycemia. However, few studies have explored this association in ICU patients. It should be noted that the pattern of blood glucose metabolism in critically ill patients was significantly different from that in diabetic patients in the community. Abnormal blood glucose is very common in ICU patients, and the stress response can increase the blood glucose of critically ill patients by regulating hormones (glucagon, catecholamine, growth hormone and cortisol) to provide enough energy for organs to survive; however, disorders of blood glucose metabolism can also introduce negative effects [1]. According to different care units and diagnostic criteria, the prevalence of abnormal glucose ranges from 30 to 60%. Age, glucose dose, insulin dose, steroid and adrenaline dose were positively related to glycemic variability in ICU patients [20]. Increased daily carbohydrate intake from enteral and parenteral nutrition led to a greater requirement for insulin and increased glycemic variability [21].

**Table 4:** Subgroup analyses for the association between CV of blood glucose and in-hospital death

Subgroups		Adjusted OR	P	P for interaction
Age	< 65 years (n = 9,197)	2.02 (1.44–2.84)	< 0.001	0.012
	≥ 65 years (n = 8,559)	1.22 (1.01–1.47)	0.042	
Race	White (n = 11,364)	1.46 (1.18–1.82)	0.001	0.208
	Other/unknown (n = 6,392)	1.24 (0.96–1.61)	0.103	
Diabetes	No (n = 12,770)	1.43 (1.17–1.74)	< 0.001	0.260
	Yes (n = 4,986)	1.21 (0.89–1.64)	0.224	
Cardiac ICU	No (n = 14,591)	1.42 (1.17–1.71)	< 0.001	0.941
	Yes (n = 3,165)	1.28 (0.87–1.88)	0.209	
Initial blood glucose	< 180 mg/dL (n = 14,201)	1.26 (1.04–1.53)	0.021	0.777
	≥ 180 mg/dL (n = 3,554)	1.36 (0.94–1.97)	0.107	

Adjusted for age, sex, ICU category, race, sepsis, myocardial infarction, heart failure, cerebrovascular disease, chronic pulmonary disease, malignant cancer, eGFR, serum sodium, potassium, mean arterial pressure, hear rate and average blood glucose

Abnormal changes in blood glucose could directly affect the function of cardiac ion channels or lead to dysfunction of cardiac autonomic nerves. The interference of cardiac repolarization and QT interval prolongation are important mechanisms of VA [8–10, 22]. Marfella et al. [23] found that the acute effect of 2 h of hyperglycemia (15 mmol/L) led to a prolonged QTc interval and increased QTc dispersion. In the experimental setting, acute hyperglycemia prolonged action potential duration (APD) in mouse ventricular myocytes, prolonged APD and induced alternation of APD in rabbit ventricular myocytes with reduced repolarization reserve [24].

Many researchers have observed that glycemic variability increased the risk of poor outcomes independent of basic blood glucose level. High glycemic variability is associated with an increased risk of all-cause mortality even when blood glucose is well controlled, suggesting that fluctuations in blood glucose could predict a residual risk of death in diabetics with well-controlled glucose status [25–27]. A recent study also found that hypoglycemia and glycemic variability were associated with mortality in ICU patients instead of hyperglycemia [28]. In addition to in-hospital mortality, in patients with acute heart failure or acute coronary syndrome admitted to the cardiac intensive unit, high glycemic variability predicted a threefold higher risk of major adverse cardiovascular events, and the cutoff value was SD > 2.7 mmol/L [29, 30]. However, no studies have attempted to relate glycemic variability, VA and in-hospital death. We found that the effect of glycemic variability on in-hospital death was achieved partly through an increased risk of VA, although the proportion was relatively low. High glycemic variability could damage multiple systems, and increase the risk of various cardiovascular disease including atrial fibrillation, heart failure and myocardial infarction, as well as brain and nerve damage, severe infection and renal dysfunction [3]. These potential mechanisms could contribute to the increased risk of in-hospital death.

In subgroup analysis from our study indicated that the association between glycemic variability and in-hospital death was more significant in patients aged < 65 years than in those older, actually, which is consistent with another study [31]. They proposed the possible mechanism is that elderly patients have increased adaptability and tolerance to damage of oxidative stress, because the level of oxidative stress naturally increase as age [32]. In addition, we assume that the elder patients have more cardiovascular disease, which could potentially conceal the effect of glycemic variability on in-hospital death.

Our findings highlight the importance of managing glycemic variability in ICU patients to reduce VA and in-hospital death. Recently, new nutrition formulae for critically ill patients were used to maintain the stability of blood glucose. A meta-analysis of randomized controlled trials demonstrated that glycemic-control formulae were superior to standard enteral formulae for reducing glycemic variability [33]. Meanwhile, a observational study indicated enteral formulae containing sustained-release starch significantly reduced glycemic variability in patients with severe acute pancreatitis and stress hyperglycemia compared with the control group [34].

The potential insights from the present study might improve more reasonable management of blood glucose and VA in critically ill patients. The present conclusion could propose that better nutritional formulations and more adequacy blood glucose control are needed to achieve the stability of blood glucose, because glycemic variability showed prognostic information independent of average blood glucose and associated with VA and in-hospital death. Meanwhile our study indicated VA is a predictor of in-hospital death related to high glycemic variability, which warn the medical stuffs in ICU setting to detect VA by continuous beside ECG monitor and provide appropriate timely treatment, such as medication, correction of electrolyte disorder, and electrical cardioversion or defibrillation. These medical interventions could reduce the incidence of in-hospital death related to blood glucose abnormalities. Notably, recent evidence has shown that sodium-glucose cotransporter 2 inhibitors (SGLT2i) have cardiovascular benefits whatever diabetic status, the improved prognosis is partly due to reduction of the arrhythmogenic effect caused by blood glucose abnormalities [35]. Therefore, the use of SGLT2i might be beneficial in reducing the risk of VA in patients with high glycemic variability.

There were several limitations in this study. Blood glucose measurements were not continuous and were unequal among patients, the severity of disease and different food intake can impact the frequency of measurement. The identification of VA was based on medical record forms, the frequency and severity of VA cannot be quantified. Hopefully, well designed prospective cohort studies with standard continuous glucose measurements and uniform ECG monitoring are done to confirm the association between glycemic variability and VA in near future.

## Conclusion

Glycemic variability was associated with an increased risk of VA and in-hospital death in ICU patients, and the effect of glycemic variability on in-hospital death was achieved partly through an increased risk of VA.

## Electronic supplementary material

Below is the link to the electronic supplementary material.

Supplementary Material 1

Supplementary Material 2

Supplementary Material 3

Supplementary Material 4

## Data Availability

MIMIC-IV database v2.0 is freely-available on PhysioNet (10.13026/7vcr-e114). The code for data query and extraction is available from the MIMIC Code Repository (https://github.com/MIT-LCP/mimic-code).

## Abbreviations

APD: action potential duration
CV: coefficient of variation
ICU: intensive care unit
IQR: interquartile range
KHB: Karlson, KB & Holm, A
MIMIC-IV: Medical Information Mart for Intensive Care IV
PVC: premature ventricular contraction
SAPS II: simplified acute physiology score II
SD: standard deviation
VA: ventricular arrhythmia
VT: ventricular tachycardia
VF: ventricular fibrillation
