# Supplementary material for: Glycemic variability and in-hospital death of critically ill patients and the role of ventricular arrhythmias
Source: Cardiovasc Diabetol. 2023 Jun 12;22:134. doi: 10.1186/s12933-023-01861-0 (PMC10258982; doi:10.1186/s12933-023-01861-0)
Supplement: Supplementary file 1 — Supplementary Material 1 [file 12933_2023_1861_MOESM1_ESM.doc]

**Supplementary materials**

Supplemental Table 1. Disease diagnosis of this study and corresponding ICD codes.

| Disease diagnosis | ICD-9 codes | ICD-10 codes |
| --- | --- | --- |
| Heart failure | 428, 39891, 40201, 40211, 40291, 40401, 40403, 40411, 40413, 40491, 40493, 4254, 4259 | I43, I50, I099, I110, I130, I132, I255, I420, I425-I429, P290 |
| Myocardial infarction | 410, 412 | I21, I22, I252 |
| Cerebrovascular disease | 438, 36234 | G45, G46, I60, I69, H340 |
| Chronic pulmonary disease | 490, 505, 4168, 4169, 5064, 5081, 5088 | J40, J47, J60, J67, I278, I279, J684, J701, J703 |
| Hypertension | 4010, 4011, 4019, 40501, 40509, 40511, 40519, 40591, 40599, 64211-64214 | I10, I15, I150-I152, I158, I159 |
| Diabetes | 2500-2509 | E100-E149 |
| Renal disease | 582, 585, 586, V56, 5880, V420, V451, 5830, 5837, 40301, 40311, 40391, 40402, 40403, 40412, 40413, 40492, 40493 | N18, N19, I120, I131, N032-N037, N052-N057, N250, Z490-Z492, Z940, Z992 |
| Malignant cancer | 140, 172, 1740, 1958, 200, 208, 2386 | C43, C88, C00-C26, C30-C34, C37-C41, C45-C58, C60-C76, C81-C85, C90-C97 |

**Supplemental Table 2. Normality test of continuous variables.**

| Variables | P for Skewness | P for Kurtosis |
| --- | --- | --- |
| Age | < 0.001 | < 0.001 |
| Heart rate | < 0.001 | 0.008 |
| Systolic BP | < 0.001 | < 0.001 |
| Diastolic BP | < 0.001 | < 0.001 |
| Mean arterial pressure | < 0.001 | < 0.001 |
| Potassium | < 0.001 | 0.2844 |
| Sodium | < 0.001 | < 0.001 |
| eGFR | < 0.001 | < 0.001 |
| Average blood glucose | < 0.001 | < 0.001 |
| CV of blood glucose | < 0.001 | < 0.001 |
| Length of ICU stay | < 0.001 | < 0.001 |
| SAPS II | < 0.001 | < 0.001 |
