# Supplementary figures and images for: Glycemic variability and in-hospital death of critically ill patients and the role of ventricular arrhythmias
Source: Cardiovasc Diabetol. 2023 Jun 12;22:134. doi: 10.1186/s12933-023-01861-0 (PMC10258982; doi:10.1186/s12933-023-01861-0)

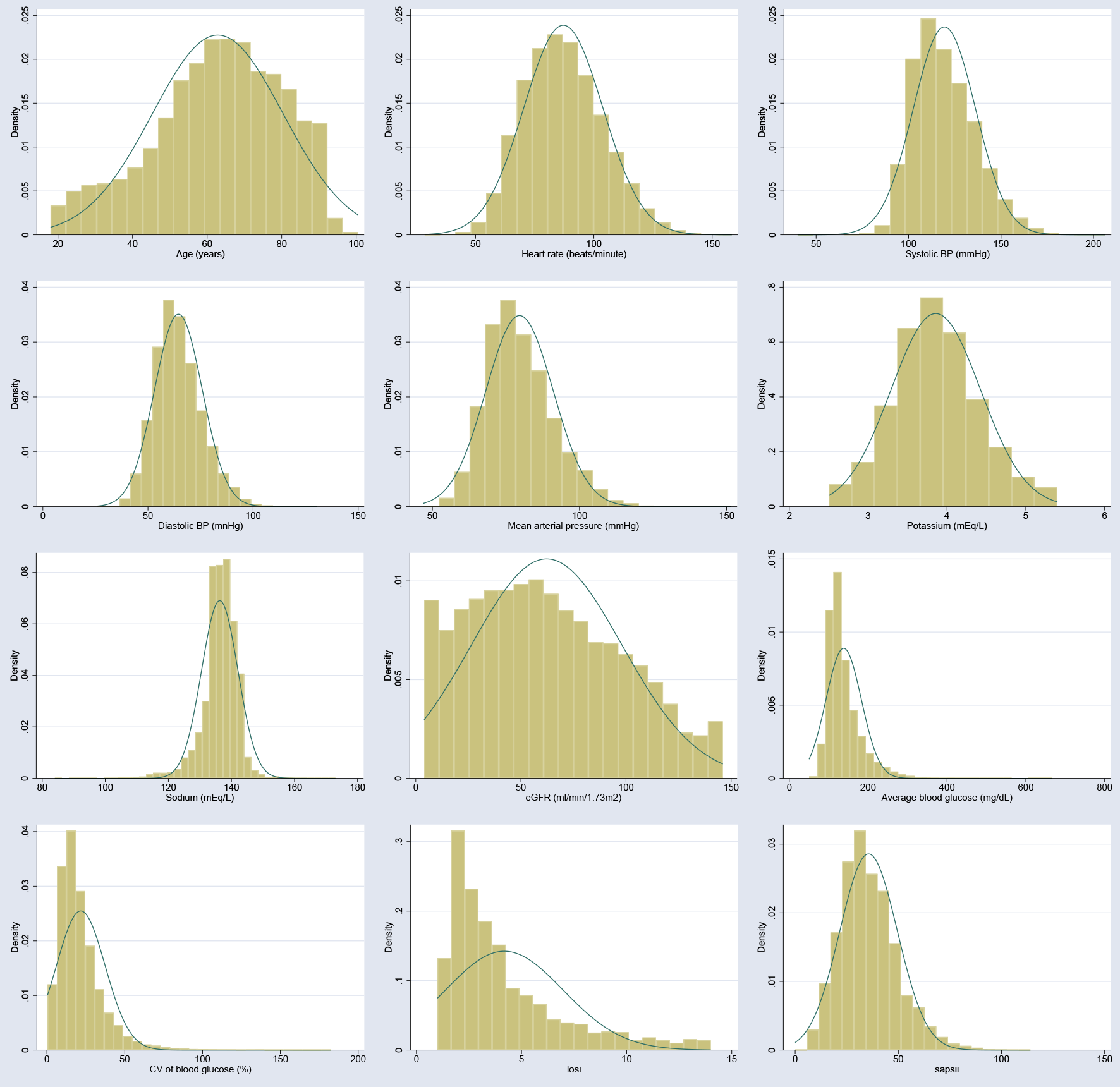

Supplement: Supplementary file 2 — Supplementary Material 2 [file 12933_2023_1861_MOESM2_ESM.tif]

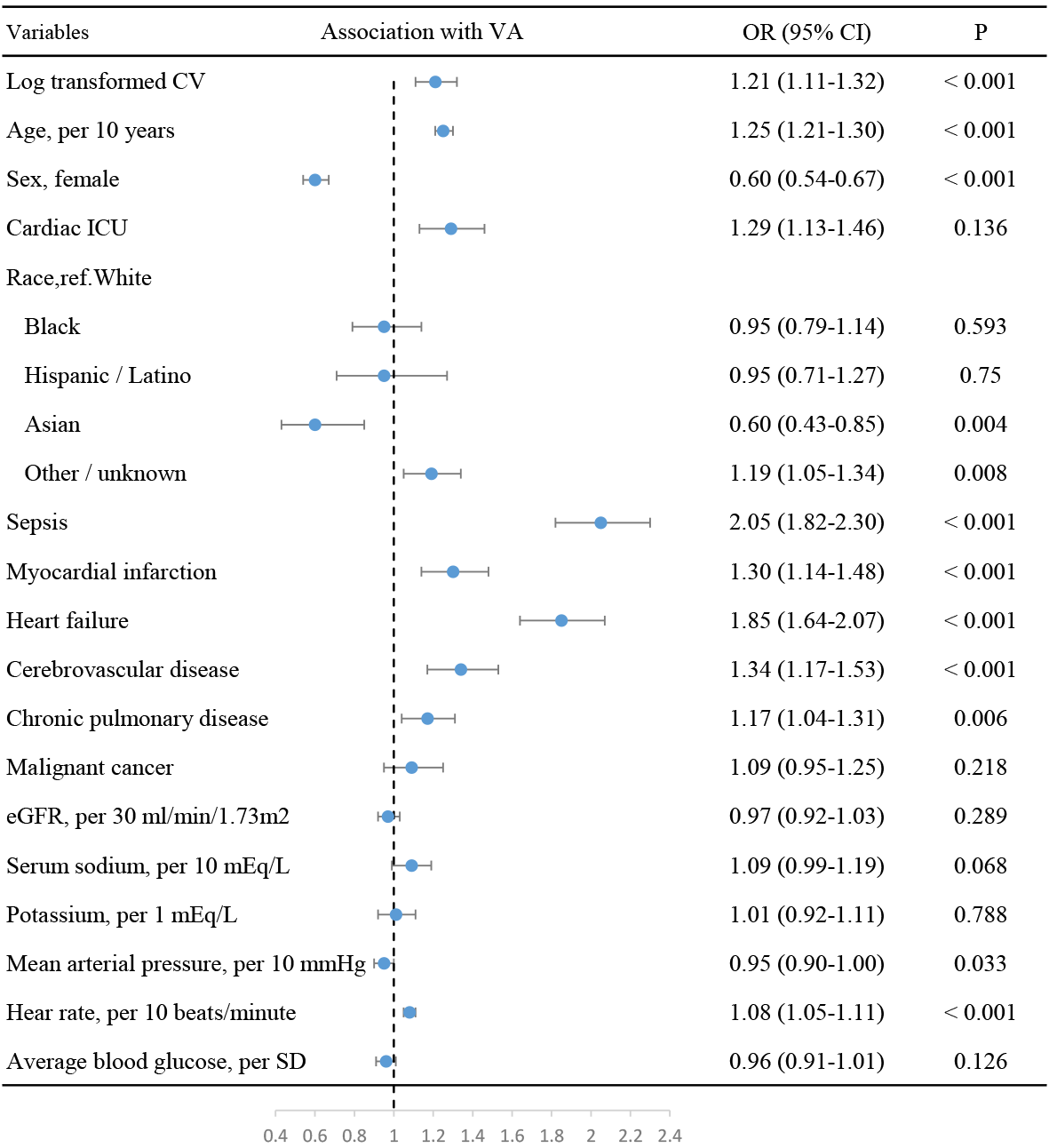

Supplement: Supplementary file 3 — Supplementary Material 3 [file 12933_2023_1861_MOESM3_ESM.tif]

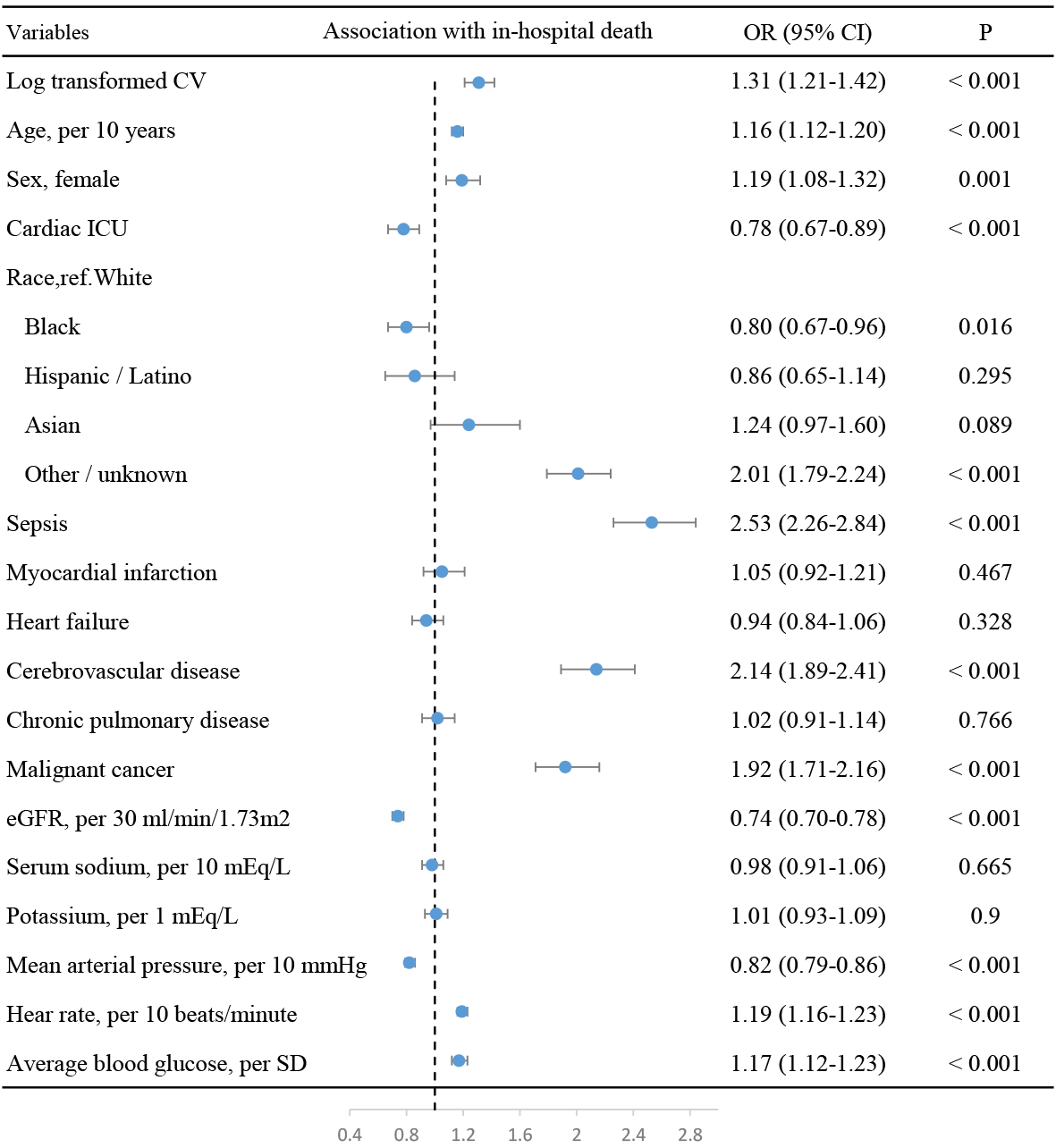

Supplement: Supplementary file 4 — Supplementary Material 4 [file 12933_2023_1861_MOESM4_ESM.tif]
